# Pharmaceutical industry payments and delivery of non-recommended and low value cancer drugs: population based cohort study

**DOI:** 10.1136/bmj-2023-075512

**Published:** 2023-10-25

**Authors:** Aaron P Mitchell, Stacie B Dusetzina, Akriti Mishra Meza, Niti U Trivedi, Peter B Bach, Aaron N Winn

**Affiliations:** 1 Department of Epidemiology and Biostatistics, Memorial Sloan Kettering Cancer Center, New York, NY 10017, USA; 2 Department of Medicine, Memorial Sloan Kettering Cancer Center, New York, NY, USA; 3 Department of Health Policy and Vanderbilt-Ingram Cancer Center, Vanderbilt University Medical Center, Nashville, TN, USA; 4 Delfi Diagnostics, Baltimore, MD, USA; 5 University of Illinois Chicago, Chicago, IL, USA

## Abstract

**Objective:**

To estimate the association between oncologists’ receipt of payments from the pharmaceutical industry and delivery of non-recommended or low value interventions among their patients.

**Design:**

Cohort study.

**Setting:**

Fee-for-service Medicare claims.

**Participants:**

Medicare beneficiaries with a diagnosis of incident cancer (new occurrence of a cancer diagnosis code in proximity to claims for cancer treatment, and no such diagnosis codes during a ≥1 year washout period) during 2014-19, who met additional requirements identifying them as at risk for one of four non-recommended or low value interventions: denosumab for castration sensitive prostate cancer, granulocyte colony stimulating factors (GCSF) for patients at low risk for neutropenic fever, nab-paclitaxel for cancers with no evidence of superiority over paclitaxel, and a branded drug in settings where a generic or biosimilar version was available.

**Main outcome measures:**

Receipt of the non-recommended or low value drug for which the patient was at risk. The primary association of interest was the assigned oncologist’s receipt of any general payments from the manufacturer of the corresponding non-recommended or low value drug (measured in Open Payments) within 365 days before the patient’s index cancer date. The two modeling approaches used were general linear model controlling for patients’ characteristics and calendar year, and general linear model with physician level indicator variables.

**Results:**

Oncologists were in receipt of industry payments for 2962 of 9799 patients (30.2%) at risk for non-recommended denosumab (median $63), 76 747 of 271 485 patients (28.3%) at risk for GCSF (median $60); 18 491 of 86 394 patients (21.4%) at risk for nab-paclitaxel (median $89), and 4170 of 13 386 patients (31.2%) at risk for branded drugs (median $156). The unadjusted proportion of patients who received non-recommended denosumab was 31.4% for those whose oncologist had not received payment and 49.5% for those whose oncologist had (prevalence difference 18.0%); the corresponding values for GCSF were 26.6% *v* 32.1% (5.5%), for nab-paclitaxel were 7.3% *v* 15.1% (7.8%), and for branded drugs were 88.3% *v* 83.5% (−4.8%). Controlling for patients’ characteristics and calendar year, payments from industry were associated with increased use of denosumab (17.5% (95% confidence interval 15.3% to 19.7%)), GCSF (5.8% (5.4% to 6.1%)), and nab-paclitaxel (7.6% (7.1% to 8.1%)), but lower use of branded drugs (−4.6% (−5.8% to −3.3%)). In physician level indicator models, payments from industry were associated with increased use of denosumab (7.4% (2.5% to 12.2%)) and nab-paclitaxel (1.7% (0.9% to 2.5%)), but not with GCSF (0.4% (−0.3% to 1.1%)) or branded drugs (1.2% (−6.0 to 8.5%)).

**Conclusions:**

Within some clinical scenarios, industry payments to physicians are associated with non-recommended and low value drugs. These findings raise quality of care concerns about the financial relationships between physicians and industry.

## Introduction

Financial relationships between US physicians and the pharmaceutical industry are common. In addition to industry research funding, US physicians overall receive more than $2bn (£1.6bn; €1.9bn) in direct, personal payments from drug and device manufacturers annually.^1^ These payments comprise both cash and in-kind gifts and most commonly represent free meals, travel and lodging, and speaker and consulting fees.^2^ Payments from industry have long raised concerns about medical professionalism and the independence of physician decision making.^3^ ^4^ ^5^ More recently, such concerns have been supported by empirical findings. Research has found a consistent association between industry payments and prescribing^6^; physicians who receive payments are more likely to prescribe the drugs manufactured by the paying company. Several studies have assessed this association using causal inference methods, strongly suggesting a causal effect of industry payments on prescribing.^7^ ^8^ ^9^

Whether industry payments to physicians have positive or negative consequences for patient care has not been evaluated empirically. The existing research examining the association between payment and prescribing has largely focused on substitution of therapeutically equivalent drugs, such as different agents within the same class. Evidence of substitution among therapeutically equivalent drugs has been found for various classes, including anticoagulants,^8^ antihypertensives,^10^ statins,^10^ ^11^ antidepressants,^10^ proton pump inhibitors,^12^ vascular endothelial growth factor inhibitors,^13^ ^14^ gabapentinoids,^15^ and cancer drugs.^16^ ^17^ In many cases, industry payments have been associated with substitution towards higher cost drugs (such as a branded drug over a same class generic), which has the potential to increase costs to the health system and to patients but does not raise immediate concerns about quality of care and patient safety. In the absence of data that raise concerns about quality, physicians’ opinions on physician-industry relationships have remained largely positive. Interview and survey studies have consistently found that physicians believe physician-industry interactions—including receipt of payments and gifts—are beneficial for patient care.^18^ ^19^ In particular, physicians view industry sponsored education and its potential to speed dissemination of new technologies into clinical practice as a rationale for physician-industry relationships to continue.^20^ ^21^ ^22^

The question of whether industry payments to physicians may be associated with lower value or non-recommended care is particularly relevant to oncology. The prevalence of industry payments is higher among oncologists than among other specialties, implying a greater opportunity to influence cancer care. Oncologists are among the top three medical specialties for dollars accepted from industry per capita,^23^ and the gap between oncologists and general internists has increased in recent years.^24^ Additionally, the high prices of cancer drugs mean that low value use is particularly costly, and the greater treatment toxicity that is commonly accepted among cancer drugs creates the potential for non-recommended treatments to be more directly harmful. In this study we assessed the association between industry payments and the delivery of non-recommended and low value interventions.

## Methods

### Non-recommended and low value interventions

Cancer interventions that were either not recommended (discouraged by guidelines) or of low value (providing no incremental benefit while being more expensive) were identified (see table 1). We included interventions where the patient group for which the intervention would not be recommended or would be of low value would be identifiable using claims data, and the relevant manufacturer commonly made payments to oncologists related to that intervention during 2014-19. Open Payments data indicate which drug or device each industry payment was associated with; among oncologists, payments associated with branded pharmaceutical products are common, whereas those associated with generic drugs and imaging modalities are rare.

**Table 1:** Non-recommended and low value drugs included in the study

Cancer drugs*	Basis for inclusion
**Non-recommended**	
Denosumab for castration sensitive prostate cancer	CALGB 90202,^25^ STAMPEDE,^26^ NCCN guidelines,^28^ expert opinion^31^ ^32^
Granulocyte colony stimulating factors with chemotherapy that is low risk for neutropenic fever	NCCN guidelines,^33^ ASCO Choosing Wisely,^34^ ASCO guidelines^35^
**Low value**	
Nab-paclitaxel for breast or lung cancer	NCT00540514,^36^ CALGB 40502^37^ ^38^
Branded drug when generic or biosimilar version is available†	NA

ASCO=American Society of Clinical Oncology; CALGB=Cancer and Leukemia Group B trial; NA=not applicable; NCCN=National Comprehensive Cancer Network; NCT=national clinical trial; STAMPEDE=Systemic Therapy in Advancing or Metastatic Prostate Cancer: Evaluation of Drug Efficacy.

* Clinical trial evidence, clinical practice guidelines, and expert opinion applied in identifying the service as non-recommended or love value is presented.

† Based on assumption that generic and biosimilar drugs are therapeutically equivalent and have lower prices than branded drugs.

Four drug interventions met these requirements: two non-recommended and two low value drugs. The first non-recommended drug was denosumab for castration sensitive prostate cancer. For bone modifying agents for castration sensitive prostate cancer, two phase 3 randomized trials found no reduction in skeletal related events from zoledronic acid,^25^ ^26^ ^27^ and denosumab has not been evaluated in the castration sensitive prostate cancer setting. Treatment with bone modifying agents is therefore not recommended for patients with castration sensitive prostate cancer,^28^ ^29^ ^30^ and such treatment is recognized as medical overuse.^31^ ^32^ The second non-recommended drug was granulocyte colony stimulating factors (GCSF) in primary prophylaxis of neutropenic fever among patients receiving chemotherapy that confers low (<10%) risk of neutropenic fever, which the National Comprehensive Cancer Network and American Society of Clinical Oncology recommend against.^33^ ^34^ ^35^ The low value drugs were nab-paclitaxel instead of paclitaxel for patients with breast or lung cancer, for which nab-paclitaxel confers no additional benefit,^36^ ^37^ ^38^ and use of a branded cancer drug or biologic agent when generic or biosimilar versions are available.

### Patient sample

Research for this paper was based on Medicare data maintained by FAIR Health. Using the Medicare fee-for-service claims, FAIR Health developed aggregated, summary level datasets for use by this study. In line with previous work,^39^ we identified a new diagnosis of cancer by new occurrence of a cancer related diagnosis code in proximity with claims for cancer treatment, and no previous cancer diagnosis codes or treatment claims within a minimum one year period of available Medicare claims. Individual participants were therefore observed only once in any analysis. The specific cancer diagnosis was then identified using a claims based algorithm that has previously been validated against Surveillance, Epidemiology, and End Results registry data^39^ (see supplementary material for detailed methods). We included patients with an index date (defined by the date of first cancer diagnosis code) occurring during 2014-19, to allow a sufficient baseline period to exclude prevalent cancers and outcome period for the treatments of interest. We required continuous enrollment in fee-for-service Medicare Parts A, B, and D from one year before the index date through the outcome period. From this cohort we defined four sub cohorts corresponding to each non-recommended or low value service scenario.

### Cohorts

*Castration sensitive prostate cancer*—This cohort comprised patients with incident metastatic prostate cancer who received cancer directed treatment and lacked evidence of an appropriate indication for bone modifying agents (eg, osteoporosis). The outcome in this cohort was any claim for denosumab within 180 days of the index date.

*GCSF*—This cohort comprised patients with incident breast, lung, or colorectal cancer who within 180 days of the index date received a chemotherapy regimen with <10% risk of neutropenic fever (see supplementary table 1).^40^ ^41^ ^42^ The outcome in this cohort was any claim for growth factor treatment from one day before to seven days after the date of a first chemotherapy claim, as done previously.^41^

*Nab-paclitaxel*—This cohort comprised patients with incident breast or lung cancer who received either nab-paclitaxel or paclitaxel. The outcome in this cohort was the first claim for either drug (nab-paclitaxel or paclitaxel) being for nab-paclitaxel.

*Branded drug*—This cohort comprised patients with several cancer types commonly treated with an agent for which a generic or biosimilar version first became available during the 2014-19 study period (imatinib, bortezomib, erlotinib, bevacizumab, trastuzumab, rituximab, and abiraterone), and with an index date that was subsequent to the availability of a generic or biosimilar agent (see supplementary table 2). The outcome in this cohort was the first claim for the drug of interest being the branded version rather than a generic or biosimilar agent.

### Receipt of industry payments

We identified the primary oncologist for each patient using claims. Briefly, we included claims from the Inpatient, Outpatient, Carrier, and Durable Medical Equipment files and assigned patients to the physician (identified by unique national provider identifier) with an associated Medicare specialty code related to oncology who had the plurality of evaluation and management claims during the period of 30 days before to 60 days after the index date.^43^

Each patient’s assigned physician was mapped to Open Payments by name and practice address.^17^ ^44^ We identified industry payments occurring during the 365 days before each patient’s index date; we defined receipt of industry payment as any industry payment for the drugs of interest (as reported by the paying pharmaceutical manufacturer) during that period. This definition allows patients assigned to the same physician to have different payment statuses, depending on the individual patients’ index dates with respect to the physician’s payment history. The drugs of interest for each cohort were those that defined the primary outcome, such as denosumab for the castration sensitive prostate cancer cohort (see supplementary table 3).

### Statistical analysis

The distribution of payments among oncologists was assessed using descriptive statistics. We report data as means and medians and as interquartile ranges expressed as a single value (ie, quartile 3 minus quartile 1, rather than the values of quartile 3 and quartile 1 separately).

Fisher’s exact test was used to assess differences in the unadjusted prevalence of non-recommended or low value drugs. We assessed the association of non-recommended and low value drugs with industry payments using two modeling approaches. In the first approach, the patient characteristics model was a linear probability model with adjustment for calendar year, age, comorbidity, and zip code level median income^45^ ^46^; we also fit analogous logistic regression models to produce estimates on the relative scale. The second approach used physician level indicator variables, also known as disjoint variables or fixed effects, to account for all time invariant characteristics of the physicians, some of which may be associated with both receipt of industry payments and care delivery (eg, physicians’ personal characteristics, specialization, practice setting, and a general propensity to both use low value drugs and accept industry payments). The inclusion of physician indicators allows for comparison of outcomes within an individual physician whose receipt of industry payments varied across time; physicians assigned only one patient in the cohort would not contribute to the estimated association, and a statistically significant association would be observed only if individual physicians delivered care differently when receiving versus not receiving payments. We estimated robust standard errors to account for physician level clustering. Linear probability models rather than non-linear models were used because they are less likely to produce biased estimates when applying a large number of indicator variables.^46^

In a separate set of models, we explored dose-response to industry payments by using a multilevel definition of payments ($0, $0-99, $100-999, ≥$1000) rather than a binary definition. In a separate set of logistic regression models with year×payment interaction terms, we explored the possibility that the magnitude of an association between industry payments and non-recommended or low value drugs may vary over time. We also explored the use of non-recommended or low value drugs at physician level, grouped by previous payment from industry and prescribing behavior. Finally, we assessed whether physicians who used non-recommended or low value drugs after recently receiving industry payments tended to use these services more often even when not recently paid.

### Patient and public involvement

No patients were involved in setting the research question, developing the outcome measures, the design or implementation of the study, interpretation, or the writing of the manuscript. Our study used deidentified claims data on license from the US federal government, and privacy restrictions on these data preclude any identification or involvement of the patients whose data are included.

## Results

### Castration sensitive prostate cancer cohort

**Fig 1:**
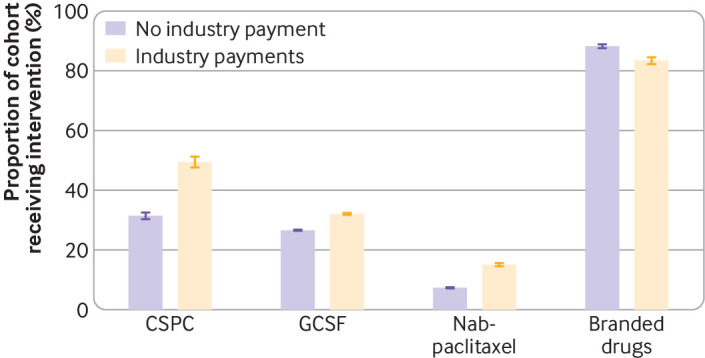
Distribution of payments from industry to oncologists and use of non-recommended or low value drugs. P<0.001 for all differences. CSPC=castration sensitive prostate cancer; GCSF=granulocyte colony stimulating factors. Whiskers represent 95% confidence intervals

The castration sensitive prostate cancer cohort included 9799 patients attributed to 5367 unique oncologists (table 2). Overall, 2962 of 9799 (30.2%) patients were attributed to oncologists who had received industry payments for denosumab within 365 days before the patient’s diagnosis (median value received $63 (interquartile range (IQR) $95)) among those with a diagnosis, this declined from 464 of 1437 (32.3%) in 2014 to 436 of 1583 (27.5%) in 2019 (table 3). Among patients whose oncologists received payments, 1466 of 2962 (49.5%) received denosumab within 180 days compared with 2150 of 6837 (31.4%) of those whose oncologists did not (prevalence difference 18.0%, P<0.001) (fig 1). In the patient characteristics model, payments from industry were associated with 17.5% (95% confidence interval 15.3% to 19.7%); odds ratio 2.07 (95% confidence interval 1.85 to 2.31)) greater denosumab usage (table 4, also see supplementary table 4). In physician level indicator models, industry payments were associated with 7.4% (95% confidence interval 2.5% to 12.2%) greater usage.

**Table 2:** Characteristics of patient cohorts and oncologists by scenario and industry payment. Values are number (percentage) unless stated otherwise

Characteristics	CSPC (n=9799)			GCSF (n=271 485)			Nab-paclitaxel (n=86 394)			Branded drug (n=13 386)	
	Paid (n=2962)	Not paid (n=6837)		Paid (n=76 747)	Not paid (n=194 738)		Paid (n=18 491)	Not paid (n=67 903)		Paid (n=4170)	Not paid (n=9216)
**Patient cohorts**											
Cancer type:											
Breast	0	0		21 816 (28.4)	54 510 (28.0)		7293 (39.4)	24 682 (36.3)		245 (5.9)	1099 (11.9)
Colon	0	0		16 482 (21.5)	25 188 (12.9)		0	0		143 (3.4)	366 (4.0)
Myeloid leukemia	0	0		0	0		0	0		678 (16.3)	796 (8.6)
Lung	0	0		33 532 (43.7)	95 696 (49.1)		11 198 (60.6)	43 221 (63.7)		<11	156 (1.7)
Myeloma	0	0		0	0		0	0		3056 (73.3)	6614 (71.8)
Non-Hodgkin’s lymphoma	0	0		0	0		0	0		<11	19 (0.2)
Prostate	2962 (100)	6837 (100)		0	0		0	0		<11	46 (0.5)
Rectum	0	0		4917 (6.4)	19 344 (9.9)		0	0		32 (0.8)	120 (1.3)
Median age (years)	76	75		72	72		72	72		73	73
Sex:											
Female	0	0		48 276 (62.9)	121 995 (62.6)		12 129 (65.6)	43 810 (64.5)		2093 (50.2)	4749 (51.5)
Male	>2950 (>99.9)*	>6820 (>99.9)*		28 439 (37.1)	72 647 (37.3)		>6340 (>34.3)*	24 066 (35.4)		2077 (49.8)	>4450 (>48.3)*
Unknown	<11	<11		32 (<0.1)	96 (<0.1)		<11	27 (0.0)		0	<11
Median zip code income ($)	59 655	62 862		58 137	61 402		58 569	60 257		60 349	62 272
Mean No of comorbidities	11.2	11.0		12.7	12.6		13.0	12.8		12.4	11.6
Index year:											
2014	464 (15.7)	973 (14.2)		15 996 (20.8)	36 457 (18.7)		4553 (24.6)	12 749 (18.8)		0	0
2015	469 (15.8)	1063 (15.5)		14 657 (19.1)	34 529 (17.7)		4058 (21.9)	12 134 (17.9)		0	0
2016	535 (18.1)	1201 (17.6)		13 578 (17.7)	32 714 (16.8)		3424 (18.5)	11 737 (17.3)		221 (5.3)	178 (1.9)
2017	504 (17.0)	1238 (18.1)		11 917 (15.5)	31 277 (16.1)		2811 (15.2)	10 756 (15.8)		214 (5.1)	215 (2.3)
2018	554 (18.7)	1215 (17.8)		11 494 (15.0)	31 094 (16.0)		2298 (12.4)	10 870 (16.0)		2093 (50.2)	3318 (36.0)
2019	436 (14.7)	1147 (16.8)		9105 (11.9)	28 667 (14.7)		1347 (7.3)	9657 (14.2)		1642 (39.4)	5505 (59.7)
**Oncologists**											
No of unique assigned oncologists	5367			18 148			14 197			7409	
No of patients per oncologist:											
Mean	1.8			15.0			6.1			1.8	
Median	1			11			4			1	
Median years since graduation	26			24			25			24	
Sex:											
Male	4158 (77.5)			11 652 (64.2)			9243 (65.1)			5214 (70.4)	
Female	1103 (20.6)			5635 (31.1)			4411 (31.1)			2172 (29.3)	
Unknown	106 (2.0)			861 (4.7)			543 (3.8)			23 (0.3)	
Located at an NCCN Institution	339 (6.3)			1844 (10.2)			1131 (8.0)			552 (7.5)	

$ 1.00 (£0.81; €0.94).

CSPC=castration sensitive prostate cancer; GCSF=granulocyte colony stimulating factors; NCCN=National Comprehensive Cancer Network.

* Exact numbers masked to avoid identification.

**Table 3:** Distribution of industry payments by cohort. Values are number (percentage) unless stated otherwise

	CSPC (n=9799)	GCSF (n=271 485)	Nab-paclitaxel (n=86 394)	Branded drug (n=13 386)
Oncologist received payment during 365 days before index date	2962 (30.2)	76 747 (28.3)	18 491 (21.4)	4170 (31.2)
No of payments among those who received any payments:				
Mean	6.3	6.1	6.5	12.9
Median (IQR)	4 (6)	4 (6)	4 (6)	7 (16)
$ value of payments among those who received any payments:				
Mean	589	219	1356	1267
Median (IQR)	63 (95)	60 (83)	89 (182)	156 (301)
Oncologist received payment during 365 days before index date, by calendar year of index date:				
2014	464 (32.3)	15 996 (30.5)	4553 (26.3)	0
2015	469 (30.6)	14 657 (29.8)	4058 (25.1)	0
2016	535 (30.8)	13 578 (29.3)	3424 (22.6)	221 (55.4)
2017	504 (28.9)	11 917 (27.6)	2811 (20.7)	214 (49.9)
2018	554 (31.3)	11 494 (27.0)	2298 (17.5)	2093 (38.7)
2019	436 (27.5)	9105 (24.1)	1347 (12.2)	1642 (23.0)

$ 1.00 (£0.81; €0.94).

CSPC=castration sensitive prostate cancer; GCSF=granulocyte colony stimulating factors; IQR=interquartile range.

**Table 4:** Association between industry payments and non-recommended or low value drugs. Associations are shown in terms of prevalence difference, paid minus not paid

Cohort	No of patients	No of oncologists	Unadjusted prevalence difference (%)	Estimated prevalence difference, % (95% CI)	
				Patient characteristics model*	Physician indicator model
CSPC	9799	5367	18.0	17.5 (15.3 to 19.7)	7.4 (2.5 to 12.2)
GCSF	271 485	18 148	5.5	5.8 (5.4 to 6.1)	0.4 (−0.3 to 1.1)
Nab-paclitaxel	86 394	14 197	7.8	7.6 (7.1 to 8.1)	1.7 (0.9 to 2.5)
Branded drugs	13 386	7409	−4.8	−4.6 (−5.8 to −3.3)	1.2 (−6.0 to 8.5)

CI=confidence interval; CSPC=castration sensitive prostate cancer; GCSF=granulocyte colony stimulating factors.

* Accounts for calendar year and patients’ characteristics.

### GCSF cohort

The GCSF cohort comprised 271 485 patients attributed to 18 148 unique oncologists (table 2). Overall, 76 747 of 271 485 (28.3%) patients were attributed to oncologists who had received industry payments for GCSF (median $60 (IQR $83), declining from 15 996 of 52 453 (30.5%) in 2014 to 9105 of 37 772 (24.1%) in 2019 (table 3). Among patients whose oncologists received payments, 24 637 of 76 747 (32.1%) received GCSF during their first cycle of low risk chemotherapy compared with 51 865 of 194 738 (26.6%) of those whose oncologist did not (prevalence difference 5.5%, P<0.001) (fig 1). In the patient characteristics model, industry payment was associated with 5.8% (95% confidence interval 5.4% to 6.1%); odds ratio 1.33 (95% confidence interval 1.28 to 1.38)) greater GCSF usage (table 4, also see supplementary table 4). In physician level indicator models, industry payments were not associated with GCSF usage (0.4% (95% confidence interval −0.3% to 1.1%)).

### Nab-paclitaxel cohort

The nab-paclitaxel cohort comprised 86 394 patients attributed to 14 197 unique oncologists (table 2). Overall, 18 491 of 86 394 (21.4%) patients were attributed to oncologists who had received industry payments for nab-paclitaxel (median $89 (IQR $182)), declining from 4553 of 17 302 (26.3%) in 2014 to 1347 of 11 004 (12.2%) in 2019 (table 3). Among patients whose oncologists received payments, 2788 of 18 491 (15.1%) received nab-paclitaxel rather than paclitaxel, compared with 4973 of 67 903 (7.3%) of those whose oncologists did not receive payments (prevalence difference 7.8%, P<0.001) (fig 1). In the patient characteristics model, industry payments were associated with 7.6% (95% confidence interval 7.1% to 8.1%); odds ratio 2.21 (95% confidence interval 2.06 to 2.38)) greater nab-paclitaxel usage (table 4, also see supplementary table 4). In physician level indicator models, industry payments were associated with 1.7% (95% confidence interval 0.9% to 2.5%) greater nab-paclitaxel usage.

### Branded drug cohort

The branded drug cohort comprised 13 386 patients attributed to 7409 unique oncologists (table 2). Overall, 4170 of 13386 (31.2%) patients were attributed to oncologists who had received industry payments for the branded drug of interest (median $156, IQR ($301)), declining from 221 of 399 (55.4%) in 2016 (the first year relevant to this cohort) to 1642 of 7147 (23.0%) in 2019 (table 3). Among patients whose oncologists received industry payments, 3480 of 4170 (83.5%) received the branded version instead of generic or biosimilar alternatives, compared with 8135 of 9216 (88.3%) among those whose oncologists did not receive payments (prevalence difference −4.8%, P<0.001) (fig 1). In the patient characteristics model, industry payments were associated with lower use of branded drugs (−4.6 (95% confidence interval −5.8 to −3.3); odds ratio 0.68 (95% confidence interval 0.61 to 0.76) (table 4, also see supplementary table 4). In physician level indicator models, industry payments were not associated with branded drug use (1.2% (95% confidence interval −6.0% to 8.5%).

Within the cohorts that comprised multiple cancer types (except for castration sensitive prostate cancer), the magnitude of the prevalence difference between paid versus not paid oncologists varied by cancer type but was generally in the same direction (supplementary table 5). Similarly, the modeled association between payments and non-recommended or low value drugs varied in magnitude among cancer types but was generally in the same direction as in the overall cohort (supplementary table 6).

In dose-response analysis, in most cases the likelihood of non-recommended or low value drugs increased with increasing payment amounts (eg, patients whose oncologist received $100-999 of payments were more likely to receive non-recommended or low value drugs than patients whose oncologist received $0-$100 of payments) (supplementary table 7). We found no evidence that the magnitude of the association between payments and non-recommended or low value drugs varied across time (supplementary table 8).

In all four cohorts, physicians who had, at least once, used a non-recommended or low value service after recently receiving an industry payment were more likely than other physicians to use non-recommended or low value cancer services even when not recently paid (supplementary table 9). However, these physicians were even more likely to use non-recommended or low value drugs when they had recently received a payment.

## Discussion

In this study, we found evidence of a patient level association between industry payments to physicians and receipt of some non-recommended and low value medical interventions. These findings are in line with previous evidence that industry payments influence physicians’ selection of therapeutically equivalent treatments,^6^ ^47^ and that there appears to be a physician level association between receipt of payments and the likelihood of using certain high cost, low value treatments.^48^ ^49^ ^50^ The finding that industry payments have the potential to be decremental, rather than neutral, may increase concern about this common practice.

Across all four cohorts in this study, the physician level indicator models produced estimates that were closer to the null than the models that adjusted for patient characteristics alone. This suggests the possibility that confounding by time invariant physician characteristics likely contributes to the magnitude of the estimated associations in the patient characteristics models. If there was an unobserved, time invariant physician characteristic that results in both a general proclivity to overuse drugs and attracts more frequent payments from industry (hypothetically, training in a particular medical specialty), this would result in an apparent positive association even in the absence of any causal impact of payments. The physician level indicator models would account for this source of confounding, and we therefore view the estimates from these models as being more robust. In these models, industry payments were associated with increased use of denosumab and nab-paclitaxel, but not GCSF or branded drugs. However, there is also an important mechanism by which the physician level indicator models may underestimate the net effect of industry payments on prescribing practices. In these models, all industry payments a physician receives that are outside of a 365 day window are absorbed as time invariant characteristics. Therefore, if, for example, a physician frequently used nab-paclitaxel because of multiple payments previously received from the drug’s manufacturer but did not further increase utilization in response to each individual payment occurring during the study period, the physician level indicator models would estimate a null association despite this causal effect. The physician level indicator models may therefore reflect the influence of individual, recent payments on care delivery but may underestimate the aggregate influence.

### Branded drug prescribing

Although industry payments were associated with several forms of non-recommended or low value drugs, they were inversely associated with branded drug prescribing in the patient characteristics model; physicians who received payment from the manufacturer of the branded drug appeared more often to prescribe the generic or biosimilar version. This is an unexpected finding, in the context of previous research suggesting industry payments may increase prescribing of branded drugs.^11^ ^15^ ^51^ ^52^ One possible explanation is confounding by practice setting. Physician practice setting (eg, academic versus community) is likely associated with both receipt of industry payments and drug selection. Industry payments are substantially more common among academically based oncologists than those in community settings.^24^ ^44^ These larger academic centers and their affiliated networks tend to be early adopters of newly available generic and biosimilar products,^53^ potentially through institution level purchasing arrangements and control of formularies. Therefore, industry payments may simply be more common among the subset of oncologists who are most likely to have institutional controls on the prescribing of branded drugs versus generic drugs. This explanation would be consistent with findings from our physician level indicator models, in which time invariant physician characteristics such as institutional setting were controlled for, and the inverse association between payments and prescribing of branded drugs was no longer observed. An additional consideration regarding the inverse association between industry payments and prescribing of branded drugs is that it was driven primarily by biosimilar agents (trastuzumab for breast cancer, bevacizumab for colon cancer, see supplementary table 5) rather than generic agents. The marketing and uptake of biosimilar agents may differ substantially from patterns seen with generic agents. In particular, manufacturers of biosimilar agents commonly make payments to physicians, whereas manufacturers of generic agents typically do not, and our study did not account for the possibility that the observed physicians may also have received payments from the competitor manufacturers of biosimilar agents.

### Potential mechanisms of the observed association

The strength of the association—and whether an association was observed—was heterogeneous. This may be related to underlying heterogeneity in the clarity and dissemination of the recommendations against these drugs. The recommendation against GCSF for low risk chemotherapy is featured in the prominent Choosing Wisely statement from the American Society of Clinical Oncology. In contrast, the National Comprehensive Cancer Network recommendations regarding denosumab for castration sensitive prostate cancer, where we observed the strongest association, are more nuanced, stating that denosumab is “similar” in effectiveness to zoledronic acid, which “is not associated with lower risk for [skeletal events]” in castration sensitive prostate cancer and “should not be used for [skeletal event] prevention until the development of metastatic CRPC [castration sensitive prostate cancer].”^28^ A separate expert consensus statement, which more clearly recommended against denosumab use in castration sensitive prostate cancer,^29^ may have lower awareness among clinicians compared with the National Comprehensive Cancer Network. As most clinicians intend to provide high quality, guideline concordant care, clear recommendations may reduce the potential for information from industry to encourage low value practices.

Industry payments, especially those of small financial value such as food and beverage payments, may have little direct impact on physician behavior but could function as markers of other kinds of interactions with industry (eg, receipt of industry information about drug products while attending sponsored meals) that are more likely to have an influence. In the mentioned case of denosumab, a plausible mechanism may be that clinicians who attend sponsored meals and thus receive more information from industry (which tends to be biased in favor of the company’s drug)^54^ may be more likely to learn about the drug’s broad approval for solid tumor malignancies from the Food and Drug Administration and less likely to learn about the established lack of benefit for castration sensitive prostate cancer.

### Clinical and policy implications

The non-recommended and low value drugs included in this study have the potential for both direct and indirect forms of patient harm. Denosumab is known to cause serious adverse events, including potentially fatal hypocalcemia and osteonecrosis of the jaw.^55^ ^56^ ^57^ Nab-paclitaxel offers no therapeutic benefit over paclitaxel in the context of breast and lung cancer but costs substantially more, contributing to the “financial toxicity” of cancer treatment and the downstream consequences of economic instability and poorer survival.^58^ ^59^

A rationale commonly presented in support of industry payments is that the information provided in conjunction with these payments improves physicians’ prescribing practice.^21^ ^60^ ^61^ In theory, by better understanding the clinical evidence and the frequency of specific toxicities, physicians will be able to select patients for treatment more appropriately. Physicians who receive industry payments would therefore be expected to have lower utilization of non-recommended and low value drugs. Our observations do not support this understanding of the role industry payments play in shaping physicians’ practice.

### Limitations of this study

This study has inherent limitations resulting from the observational, claims based design. Identifying incident cancers in claims likely results in some degree of misclassification. Physician attribution using claims is also imperfect, with the possibility of misidentifying the physician responsible for the decision about treatment, or of crediting that decision to a single physician when it was actually guided by multiple providers. Both of these sources of misclassification would be expected to bias estimates toward the null. The study was limited to specific forms of non-recommended or low value interventions that are identifiable in claims and cannot be extrapolated to other services or medical specialties. We observed a decrease in the prevalence of industry payments for the non-recommended or low value drugs (table 3), consistent with previous observations that payments from industry for individual drugs are greatest at initial approval and subsequently decline,^62^ which may mitigate any effect of payments over time. Our dataset was limited in the range of observable patient and physician level characteristics available for adjustment. We addressed this concern about physicians’ characteristics by applying physician level indicator variables, which absorb all time invariant physician characteristics, both observed and unobserved. Regarding patient characteristics, we anticipated that age and comorbidity count would be the characteristics most likely to impact the delivery of care; the observation that adjustment for these characteristics had little impact on the unadjusted estimates (table 4) suggests minimal confounding by these characteristics. This study can conclude only an association between industry payments and prescribing and cannot infer causality. Though the non-recommended and low value drugs we studied have the potential to harm patients through several mechanisms, further research will be needed to evaluate the association between industry payments and patient outcomes.

### Conclusions

The influence of industry payments on physicians’ behavior is well established. This study suggests that this influence has the potential to negatively impact the care of individual patients. Patients with cancer whose oncologist received payments from industry appeared more likely to receive non-recommended and low value treatments. This study focused on a narrow group of patients and interventions, and further research is needed to better characterize whether, and to what degree, the observed association between payments and poorer care quality extends to other settings. Given the potential concerns for care quality raised by this study, however, it may be appropriate to re-examine the current status of personal payments from the drug industry to physicians.

**What is already known on this topic**

A systematic review found a statistically significant, positive association between receipt of payments from the drug industry and physicians’ prescribing behavior in all of the 36 included studies
The several included studies that applied causal inference methods supported a causal effect of payments on prescribing
The included studies measured changes in prescribing among medically appropriate or interchangeable drugs, not whether receipt of industry payments may be associated with non-recommended or low value drugs

**What this study adds**

Patients with cancer whose oncologist received payments from industry appeared more likely to receive non-recommended and low value treatments
This finding raises potential concerns about quality of care

## Data Availability

Data analysis was conducted under contract agreement with FAIR Health, a Centers for Medicare and Medicaid Services qualified entity. The authors are solely responsible for the research and conclusions reflected in this paper. FAIR Health is not responsible for the conduct of the research or for any of the opinions expressed in this paper. APM had full access to all of the aggregated, deidentified datasets used in the study and takes responsibility for the integrity of the data and the accuracy of the data analysis.

## Web extra

Extra material supplied by authors

## Notes

The lead author (APM) affirms that the manuscript is an honest, accurate, and transparent account of the study being reported; that no important aspects of the study have been omitted; and that any discrepancies from the study as originally planned (and, if relevant, registered) have been explained.

Dissemination to participants and related patient and public communities: The results of this work will be disseminated to the public through institutional press release, ensuing news articles, social media, and an opinion piece authored by the study’s authors that describe the study’s findings for the public.

Provenance and peer review: Not commissioned; externally peer reviewed.
