# Supplementary material for: Pharmaceutical industry payments and delivery of non-recommended and low value cancer drugs: population based cohort study
Source: BMJ. 2023 Oct 25;383:e075512. doi: 10.1136/bmj-2023-075512 (PMC10599253; doi:10.1136/bmj-2023-075512)
Supplement: Supplementary file 1 — Supplementary information: Additional methods, figure, and tables -9 [file mita075512.ww.pdf]

Pharmaceutical Industry Payments and Delivery of Non-Recommended and Low-Value Cancer Care Services: A Cohort Study

SUPPLEMENTAL APPENDIX

Supplementary Methods………………………………………………………………..…p.2
Supplementary Figure: Cohort definition……………………………………………………p.4
Supplementary Table 1: Chemotherapy regimens with low risk for neutropenic fever……….p.5
Supplementary Table 2: Cancer drugs included within the branded drug cohort……..….……p.6
Supplementary Table 3: Drugs used to define exposure to industry payments……….……….p.7
Supplementary Table 4: Exposure/outcome distribution within individual cancer types...…….p.8
Supplementary Table 5: Association between industry payments and non-recommended/low-value services within individual cancer types……………………….…………………….p.9
Supplementary Table 6: Association between industry payments and non-recommended/low-value services within individual cancer types………………………………………………p.10
Supplementary Table 7: Dose-responsiveness of non-recommended/low-value services to dollar value of payments…………………………………………………………………………...p.11
Supplementary Table 8: Analysis of time trends in the association between industry payments and use of non-recommended/low-value services…………………………………………..p.12
Supplementary Table 9: Use of non-recommended/low-value services among physicians grouped by industry payment and prescribing history……………………………………………..p.13

Supplementary Methods, cohort creation

Eligible Medicare beneficiaries were required to meet one of the following criteria:
1) A claim in the Inpatient file containing a principal or other diagnosis of cancer.
2) A claim in the Outpatient file having both: (1) a principal or other diagnosis of cancer; and (2) a HCPCS code mapping to a former Berenson-Eggers Type of Service (BETOS) code associated with cancer treatment.
3) A claim in the Carrier file having both: (1) a principal or other diagnosis of cancer; and (2) a BETOS code associated with cancer treatment.
4) A claim in the DME file having both: (1) a principal or other diagnosis of cancer; and (2) an NDC mapping to an oral cancer drug.
5) An event in the Part D event data file mapping to an NDC of an oral cancer drug.

We included those with an index date (defined by the date of first cancer diagnosis code) occurring in 2014-2019. If a beneficiary had a claim which met criteria 1-5 above, but occurred between 1/1/2013 and the index date, they were excluded; this step was to avoid inclusion of beneficiaries with prevalent cancers. All beneficiaries were required to have continuous enrollment in fee-for-service Medicare Parts A, B, D from >= 365 days prior to their index date through the end of the cohort-specific outcome period (see below). Those who were not attributable to a unique oncologist were excluded.

To define the cancer site for each beneficiary, we applied a hierarchical algorithm evaluating all claims occurring from index date through 180 days afterwards. In summary:
1) For beneficiaries with any inpatient claims, they were assigned the cancer site with the highest number of inpatient claims (or, in cases of ties, to the cancer site with claims totaling the highest payment amount).
2) For beneficiaries with any hospital outpatient claims, they were assigned the cancer site with the highest number of hospital outpatient claims (or, in cases of ties, to the cancer site with claims totaling the highest payment amount).
3) For beneficiaries with physician office claims only, they were assigned the cancer site with the highest number of physician office claims (or, in cases of ties, to the cancer site with claims totaling the highest payment amount).

Cohort-specific requirements

Castrate-sensitive prostate cancer (CSPC) cohort

1) Beneficiaries were excluded for any occurrence of claims with an associated diagnosis code for osteoporosis, osteopenia, hypercalcemia, bone fracture, or any claim for a bisphosphonate drug or denosumab prior to index date.
2) Beneficiaries were required to have presence of bone metastasis, was defined by 1 or more inpatient claims with a primary or secondary diagnosis code of bone metastasis OR 2 or more outpatient claims for treatments, diagnostic procedures, or evaluation & management with diagnosis codes for bone metastasis, occurring between 30 days prior to index date and 180 days after index date.
3) Beneficiaries were required to have a claim for androgen deprivation therapy (leuprolide, goserelin, triptorelin, abarelix, cetrorelix, ganirelix, degarelix, or surgical orchiectomy) or an

antiandrogen drug (bicalutamide, flutamide, or nilutamide) occurring between 30 days prior to index date and 180 days after index date.
4) Beneficiaries were excluded for diagnosis of osteoporosis, osteopenia, hypercalcemia, or bone fracture occurring between index date and 180 days thereafter.

Granulocyte colony stimulating factor (GCSF) cohort
1) Beneficiaries were required to have first chemotherapy claim within 180 days of index date.
2) A list of chemotherapy regimens with low (<10%) risk of neutropenic fever was compiled from previously published studies and guidelines (**Supplementary Table 1**).[33–35]
3) To be categorized as having received one of these regimens, we looked forward 30 days from the first chemotherapy claim. Beneficiaries were required to have received no other chemotherapy drugs besides the drug (or drugs) that comprised the regimen. In cases of multi-drug regimens, we also required that claims for each of the constituent drugs occur on the first day with any chemotherapy claims. Beneficiaries that did not meet these requirements to be categorized as having received low-risk chemotherapy were excluded.

Nab-paclitaxel cohort
1) Beneficiaries with incident breast or lung cancer were included.
2) Beneficiaries were included if they had any claims for either paclitaxel or nab-paclitaxel within 365 days of index date; all those with no claims for either drug within 365 days were excluded.

Branded drug cohort
1) Beneficiaries with incidence of any of the cancer types treated with one (or more) of the identified list of drugs with new generic or biosimilar competition (**Supplementary Table 2**) were included.
2) Beneficiaries were excluded if their index date occurred prior to when the relevant generic/biosimilar competitor entered the market (eg., prior to 1/1/2018 for multiple myeloma).
3) Beneficiaries were included if they had any claims for the drug of interest corresponding to their cancer type (either the branded or the generic/biosimilar version) within 365 days of index date; all those with no claims within 365 days were excluded.

Supplementary Figure: Cohort definition. In the final step, indicated by a differently colored arrow for each cohort, eligible patients are drawn from several cancer types according to cohort-specific criteria as described in Supplementary Methods. CSPC, castration sensitive prostate cancer; GCSF, granulocyte colony stimulating factor; CML, chronic myeloid leukemia; NHL, non Hodgkin lymphoma.

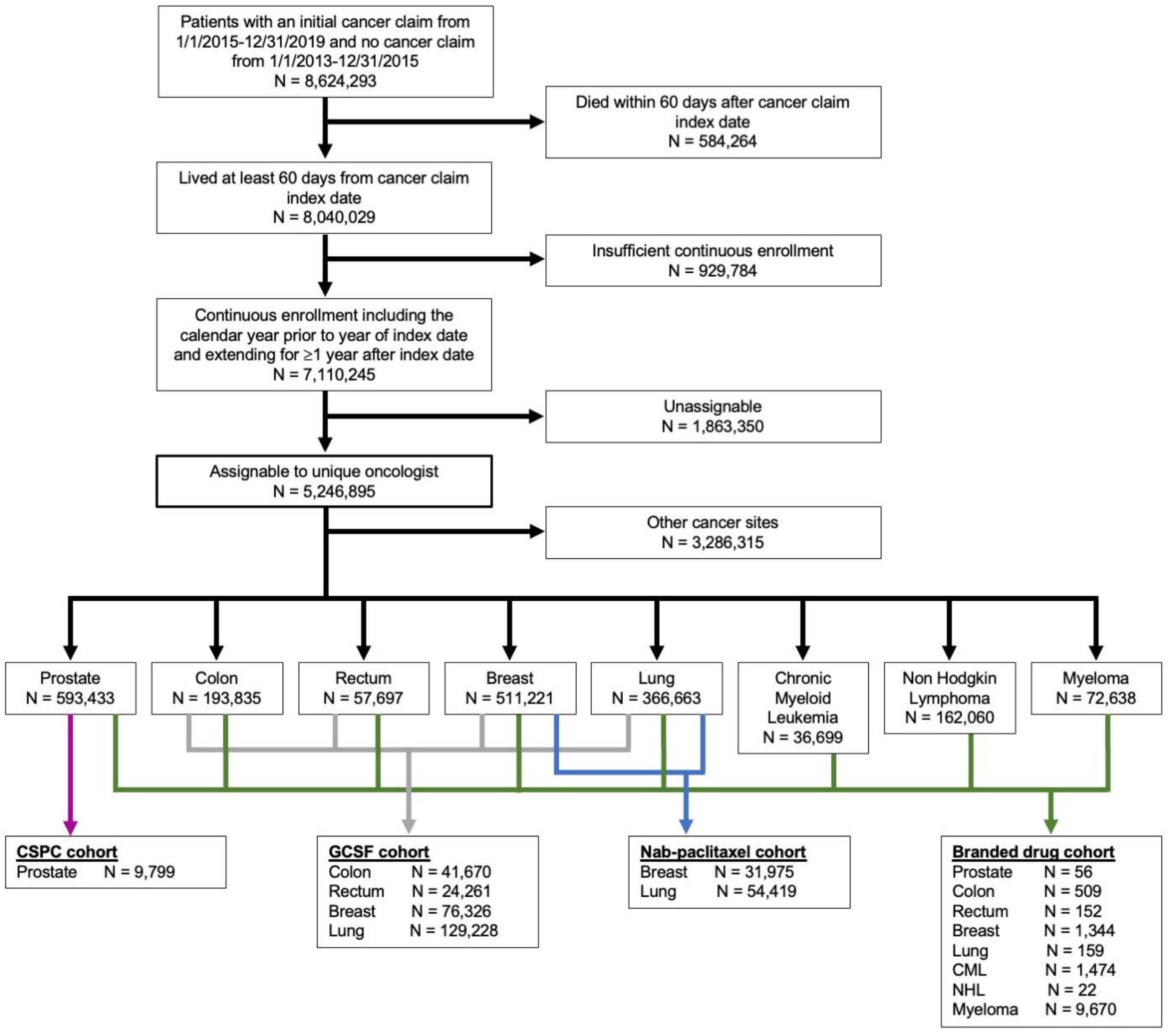

Supplementary Table 1: **Chemotherapy regimens with low risk for neutropenic fever.** Sources included previously-published studies and clinical practice guidelines. The treatment regimen carboplatin + paclitaxel was excluded because there was disagreement between sources regarding whether its neutropenic fever risk was low.

**Cancer type**	**Regimen**
Breast	CMF (cyclophosphamide + methotrexate + fluorouracil)
	EC (epirubicin + cyclophosphamide)
	Liposomal doxorubicin
	Capecitabine
	Gemcitabine
	Vinorelbine
	Eribulin
	Cyclophosphamide
	Carboplatin
	Cisplatin
	Epirubicin
	Ixabepilone
	Gemcitabine + carboplatin
	Paclitaxel + bevacizumab
	Paclitaxel + trastuzumab
	Trastuzumab + vinorelbine
	Trastuzumab + capecitabine
Lung	Cisplatin + pemetrexed
	Carboplatin + gemcitabine
	Cisplatin + gemcitabine
	Carboplatin + pemetrexed
	Cisplatin + vinblastine
	Any low-risk doublet + (bevacizumab, pembrolizumab, or atezolizumab)
	Gemcitabine + vinorelbine
	Nab-paclitaxel
	Gemcitabine
	Paclitaxel
	Pemetrexed
	Vinorelbine
Colon, rectum	5-fluorouracil + leucovorin
	5-fluorouracil
	Capecitabine
	Bevacizumab
	Cetuximab
	Panitumumab

Supplementary Table 2: **Cancer drugs included within the branded drug cohort.**

Cancer type[s] included	Drug, branded name	Drug, generic or biosimilar name	First generic availability date
Chronic myeloid leukemia	Gleevec	imatinib	2/1/16
Multiple myeloma	Velcade	bortezomib	1/1/18
Lung	Tarceva	erlotinib	5/1/19
Lung, colon, rectal	Avastin	bevacizumab-awwb (Mvasi), bevacizumab-bvzr (Zirabev)	7/1/19
Breast	Herceptin	trastuzumab-anns (Kanjinti), trastuzumab-qyyp, trastuzumab-dttb, trastuzumab-pkrb, trastuzumab-dkst (Ogivri)	7/1/19
Non-Hodgkin lymphoma	Rituxan	rituximab-abbs (Truxima), rituximab-pvvr (Ruxience), rituximab-arrx (Riabni)	11/1/19
Prostate	Zytiga	abiraterone	11/1/19

Supplementary Table 3: Drugs used to define exposure to industry payments. Open Payments files were searched for payment events wherein the associated "Name of Drug…" field corresponded to the drug[s] of interest for that cohort. The manufacturer of each drug of interest is shown but was not used directly in the analysis.

**Cohort**	**Drug of interest (Brand name)**	**Manufacturer**
CSPC	Denosumab (Xgeva)	Amgen
GCSF	Filgrastim (Neupogen)<br>Filgrastim-sndz (Zarxio)<br>Filgrastim-aafi (Nivestym)<br>Peg-filgrastim (Neulasta)<br>Pegfilgrastim-jmdb (Fulphila)<br>Pegfilgrastim-cbqv (Udenyca)<br>Pegfilgrastim-bmez (Ziextenzo)<br>Pegfilgrastim-apgf (Nyvepria)	Amgen<br>Novartis<br>Pfizer<br>Amgen<br>Mylan<br>Coherus<br>Sandoz<br>Pfizer
Nab-paclitaxel	Nab-paclitaxel (Abraxane)	Celgene
Branded drugs	Imatinib (Gleevec)<br>Bortezomib (Velcade)<br>Rituximab (Rituxan)<br>Abiraterone (Zytiga)<br>Erlotinib (Tarceva)<br>Bevacizumab (Avastin)<br>Trasuzumab (Herceptin	Novartis<br>Takeda<br>Genentech<br>Janssen<br>Genentech<br>Genentech<br>Genentech

Supplementary Table 4: Estimates of the association between industry payments and use of non-recommended/low-value services on the relative scale. Estimates are from logistic regression models with adjustment for calendar year, patient age, patient comorbidity, and ZIP code median income, and with standard errors clustered at the physician level.

**Cohort**	**OR (95% CI)**
CSPC	2.07 (1.85 to 2.31)
GCSF	1.33 (1.28 to 1.38)
Nab-paclitaxel	2.21 (2.06 to 2.38)
Branded drugs	0.68 (0.61 to 0.76)

Supplementary Table 5: **Exposure/outcome distribution within individual cancer types.**

Cancer type	Total	No payments, outcome -	No payments, outcome +	Yes payments, outcome -	Yes payments, outcome +	Outcome prevalence, unexposed	Outcome prevalence, exposed	Prevalence difference
**CSPC cohort**								
All	9,799	4,687	2,150	1,496	1,466	31.4%	49.5%	18.0%
Prostate	9,799	4,687	2,150	1,496	1,466	31.4%	49.5%	18.0%
**GCSF cohort**								
All	271,485	142,873	51,865	52,110	24,637	26.6%	32.1%	5.5%
Breast	76,326	22,793	31,717	8,663	13,153	58.2%	60.3%	2.1%
Colon	41,670	23,771	1,417	14,842	1,640	5.6%	10.0%	4.3%
Lung	129,228	77,445	18,251	23,976	9,556	19.1%	28.5%	9.4%
Rectum	24,261	18,864	480	4,629	288	2.5%	5.9%	3.4%
**Nab-paclitaxel cohort**								
All	86,394	62,930	4,973	15,703	2,788	7.3%	15.1%	7.8%
Breast	31,975	23,511	1,171	6,700	593	4.7%	8.1%	3.4%
Lung	54,419	39,419	3,802	9,003	2,195	8.8%	19.6%	10.8%
**Branded drugs cohort**								
All	13,386	1,081	8,135	690	3,480	88.3%	83.5%	-4.8%
Breast	1,344	165	934	51	194	85.0%	79.2%	-5.8%
Colon	509	49	317	34	109	86.6%	76.2%	-10.4%
Myeloid Leukemia	1,474	672	124	543	135	15.6%	19.9%	4.3%
Lung	*	27	129	*	*	82.7%	N/A	N/A
Multiple Myeloma	9,670	95	6,519	47	3,009	98.6%	98.5%	-0.1%
Non-Hodgkin lymphoma	*	18	*	*	*	N/A	N/A	N/A
Prostate	*	39	*	*	*	N/A	N/A	N/A
Rectum	*	16	104	*	29	86.7%	N/A	N/A

*Cell suppressed to prevent re-identification

Supplementary Table 6: Association between industry payments and non-recommended/low-value services within individual cancer types. For each cohort comprising patients with more than one cancer type, model results (both patient characteristics model and physician indicator model) are shown overall and within each cancer type. For the branded drug cohort, only the multiple myeloma subgroup is shown because the small sample sizes for the other cancer types resulted in model non-convergence.

		**Estimated prevalence difference with exposure to industry payments, % (95% CI)**	
**Cohort**	**Patients, No.**	**Patient characteristics model**	**Physician indicator model**
GCSF, overall	271,485	5.8 (5.4 to 6.1)	0.4 (-0.3 to 1.1)
GCSF, lung	129,228	9.4 (8.8 to 9.7)	0.8 (-0.1 to 1.8)
GCSF, colon	41,670	4.4 (3.9 to 4.9)	0.6 (-0.3 to 1.5)
GCSF, rectum	24,261	3.4 (2.9 to 4.0)	0.8 (-0.2 to 2.0)
GCSF, breast	76,326	2.8 (2.1 to 3.6)	-0.5 (-2.0 to 1.0)
Nab-paclitaxel, overall	86,394	7.6 (7.1 to 8.1)	1.7 (0.9 to 2.5)
Nab-paclitaxel, lung	54,419	10.7 (10.1 to 11.4)	0.9 (-0.2 to 2.1)
Nab-paclitaxel, breast	31,975	3.2 (2.6 to 3.8)	1.9 (0.7 to 2.9)
Branded drugs, overall	13,386	-4.6 (-5.8 to -3.3)	1.2 (-5.8 to 8.3)
Branded drugs, myeloma	9,667	-0.6 (-1.1 to -0.1)	1.8 (0.3 to 3.2)

Supplementary Table 7: Dose-responsiveness of non-recommended/low-value services to dollar value of payments. Payment exposure in USD.

			Estimated prevalence difference with exposure to industry payments, % (95% CI)	
**Cohort**	**Payment exposure**	**Patient N**	**Patient characteristics model**	**Physician indicator model**
CSPC	0	6837	ref	ref
CSPC	0-99	991	11.5 (8.3 to 14.6)	4.7 (-2.3 to 10.7)
CSPC	100-999	1766	21.7 (19.2 to 24.2)	14.1 (4.8 to 23.5)
CSPC	≥1,000	205	4.8 (-1.8 to 11.4)	6.3 (-10.7 to 23.2)
GCSF	0	194,738	ref	ref
GCSF	0-99	29,681	3.7 (3.2 to 4.3)	0.6 (-0.2 to 1.3)
GCSF	100-999	45,784	7.0 (6.5 to 7.4)	0.2 (-1.2 to 1.7)
GCSF	≥1,000	1,282	10.8 (8.4 to 13.3)	-6.6 (-13.0 to -0.2)
Nab-paclitaxel	0	67,903	ref	ref
Nab-paclitaxel	0-99	7,127	3.5 (2.8 to 4.2)	1.5 (0.5 to 1.3)
Nab-paclitaxel	100-999	9,434	9.1 (8.5 to 9.7)	2.0 (0.5 to 3.4)
Nab-paclitaxel	≥1,000	1,930	15.4 (14.1 to 16.7)	2.0 (-0.1 to 5.1)
Branded drug	0	9,216	ref	ref
Branded drug	0-99	396	-10.2 (-13.5 to -6.8)	-4.0 (-22.5 to 14.6)
Branded drug	100-999	2,415	-4.3 (-5.8 to -2.8)	1.5 (-7.4 to 10.5)
Branded drug	≥1,000	1,359	-3.4 (-5.3 to -1.5)	3.9 (-11.1 to 18.8)

Supplementary Table 8: Analysis of time trends in the association between industry payments and use of non-recommended/low-value services. Estimates are from logistic regression models with adjustment for calendar year, patient age, patient comorbidity, ZIP code median income, and a calendar year*exposure interaction term. Standard errors clustered at the physician level. 2014 is the reference year in all models.

Cohort	Term	OR (95% CI)	Term	OR (95% CI)
CSPC	Exposed	2.06 (1.60 to 2.65)	-	-
	2015	1.31 (1.09 to 1.58)	2015*exposed	0.98 (0.70 to 1.35)
	2016	1.15 (0.95 to 1.38)	2016*exposed	0.91 (0.66 to 1.26)
	2017	1.04 (0.86 to 1.25)	2017*exposed	0.91 (0.66 to 1.27)
	2018	0.84 (0.69 to 1.03)	2018*exposed	1.16 (0.84 to 1.61)
	2019	0.88 (0.73 to 1.08)	2019*exposed	1.08 (0.77 to 1.51)
GCSF	Exposed	1.35 (1.28 to 1.43)	-	-
	2015	1.01 (0.97 to 1.04)	2015*exposed	1.02 (0.96 to 1.09)
	2016	1.01 (0.97 to 1.05)	2016*exposed	1.00 (0.93 to 1.07)
	2017	0.99 (0.96 to 1.03)	2017*exposed	0.94 (0.87 to 1.01)
	2018	0.98 (0.94 to 1.02)	2018*exposed	0.95 (0.88 to 1.02)
	2019	0.94 (0.91 to 0.98)	2019*exposed	0.99 (0.92 to 1.07)
Nab-paclitaxel	Exposed	2.25 (1.99 to 2.53)	-	-
	2015	0.88 (0.80 to 0.97)	2015*exposed	1.04 (0.89 to 1.21)
	2016	0.82 (0.74 to 0.91)	2016*exposed	1.00 (0.85 to 1.19)
	2017	0.64 (0.57 to 0.71)	2017*exposed	0.91 (0.75 to 1.09)
	2018	0.70 (0.63 to 0.78)	2018*exposed	0.94 (0.78 to 1.15)
	2019	0.88 (0.79 to 0.98)	2019*exposed	0.95 (0.77 to 1.17)

Supplementary Table 9: Use of non-recommended/low-value services among physicians grouped by industry payment and prescribing history. Physicians were divided into two groups, 1) those who had ever prescribed a non-recommended/low-value service to a patient included in the relevant cohort while exposed to payments (eg, when having accepted payments within the last 365 days), "paid prescribers" and 2) those who never made such prescriptions while exposed, "other oncologists." Of note, "other oncologists" may still have received industry payments and may have prescribed non-recommended/low-value services, but not within 365 days of each other. For each group of physicians, the proportions of patients who received non-recommended/low-value services during time periods when the physician had received payments in the past 365 days ("while physician was exposed") vs. when they had not ("while physician was unexposed") is shown.

	**While physician was exposed**			**While physician was unexposed**		
Cohort and physician group	Received NR/LV service (N)	Did not receive NR/LV service (N)	Percent received (%)	Received NR/LV service (N)	Did not receive NR/LV service (N)	Percent received (%)
CSPC						
Paid prescribers	1,466	483	75.2	172	180	48.9
Other oncologists	0	1,013	0	1,978	4,507	30.5
GCSF						
Paid prescribers	24,637	47,169	34.3	9,753	20,979	31.7
Other oncologists	0	4,941	0	42,112	121,894	25.7
Nab-paclitaxel						
Paid prescribers	2,788	7,008	28.5	519	2,891	15.2
Other oncologists	0	8,695	0	4,454	60,039	6.9
Branded drug						
Paid prescribers	3,480	343	91.0	638	78	89.1
Other oncologists	0	347	0	7,497	1,003	88.2
